# Does traditional asian vegetables (*ulam*) consumption correlate with brain activity using fMRI? A study among aging adults from low‐income households

**DOI:** 10.1002/jmri.26891

**Published:** 2019-08-06

**Authors:** Yee Xing You, Suzana Shahar, Mazlyfarina Mohamad, Hanis Mastura Yahya, Hasnah Haron, Hamzaini Abdul Hamid

**Affiliations:** ^1^ Dietetics Program and Center for Healthy Aging and Wellness (H‐Care), Faculty of Health Sciences Universiti Kebangsaan Malaysia, Jalan Raja Muda Abdul Aziz 50300 Kuala Lumpur Malaysia; ^2^ Diagnostic Imaging and Radiotherapy Program, Faculty of Health Sciences Universiti Kebangsaan Malaysia Kuala Lumpur Malaysia; ^3^ Nutritional Sciences Program and Center for Healthy Aging and Wellness (H‐Care), Faculty of Health Sciences Universiti Kebangsaan Malaysia Kuala Lumpur Malaysia; ^4^ Department of Radiology, Faculty of Medicine Universiti Kebangsaan Malaysia Medical Center Kuala Lumpur Malaysia

**Keywords:** brain activation, cognitive, DLPFC, fMRI, *ulam*

## Abstract

**Background:**

Working memory and cognitive flexibility are supported by the dorsolateral prefrontal cortex (DLPFC). Aging adults from low‐income households are individuals with a high risk of cognitive decline who incorporate *ulam* in their daily diet.

**Purpose:**

To examine relationship between *ulam* consumption and the working memory and cognitive flexibility among aging adults from low‐income households who are more susceptible to cognitive decline.

**Study Type:**

Cross‐sectional.

**Population/Subjects:**

Thirty‐two aging adults (45–75 years old).

**Field Strength/Sequence:**

Task‐based fMRI, 3.0T, T_1_‐weighted anatomical images, T_2_*‐weighted imaging data.

**Assessment:**

The dietary and *ulam* consumption were assessed using the respective validated Dietary History and semiquantitative Food Frequency questionnaires. Working memory and cognitive flexibility were evaluated by using neuropsychological batteries (ie, mini‐mental state examination [MMSE], Digit Span, and Rey auditory verbal learning test [RAVLT]) and task‐based fMRI (N‐back and Stroop Color Word Test [SCWT]). Brodmann's areas 9 and 46 were the regions of interest (ROIs) of DLPFC activation.

**Statistical Tests:**

Multiple linear regression used to understand the relationship between *ulam* consumption and the working memory and cognitive flexibility, while analysis of covariance (ANCOVA) was used to compare the difference of working memory and cognitive flexibility among four percentiles of *ulam* consumption, after age, gender, and education years adjustments. Significance was decided by two‐sided, *P* < 0.0042 and *P* < 0.05.

**Results:**

The multiple linear regression revealed that *ulam* consumption was positively associated with the Digit Span (R^2^ = 0.51, β = 0.702, *P* < 0.001), right DLPFC activation (1‐back) (R^2^ = 0.34, β = 0.591, *P* = 0.001), left DLPFC activation (SCWT‐1) (R^2^ = 0.33, β = 0.553, *P* = 0.002), and left DLPFC activation (SCWT‐2) (R^2^ = 0.34, β = 0.497, *P* = 0.004). The *ulam* consumption at the 75^th^ and 100^th^ percentile from the ANCOVA analysis had shown a better working memory and cognitive flexibility as compared with those of the 25^th^ and 50^th^ percentiles (*P* < 0.05).

**Data Conclusion:**

This study found that high *ulam* consumption was related to a high intensity of brain activation in DLPFC; however, the elucidation of the neuroprotective properties of *ulam* have yet to be established from clinical trial studies.

**Level of Evidence:** 2

**Technical Efficacy:** Stage 4

J. Magn. Reson. Imaging 2020;51:1142–1153.

COGNITIVE DECLINE is an indication of mental health and can lead to irreversible dementia if left untreated.^1^ As a result of the aging process,^2^ the prevalence of people with dementia in developing countries, particularly in Asia, is estimated to increase from 60% in 2001 to 71% by 2040,^3^ with more prevalent poor mental health and cognition skills observed among the lower‐income aging population in Malaysia based on the National Health Mobility Survey 2015.^4^ Working memory and cognitive flexibility are the executive functions supported by the dorsolateral prefrontal cortex (DLPFC) in human brain. Impaired synaptic neuroplasticity, which is caused by dorsolateral prefrontal cortex dysfunction, may lead to mild cognitive impairment.^5^ A recent study has successfully shown that higher vitamin B6 intake was closely related to better working memory using N‐back task functional magnetic resonance imaging (fMRI) in DLPFC among mild cognitive impairment older adults.^6^

Many Asian countries have conducted a series of studies on the prevention of neurodegenerative diseases through food‐based recommendation such as those of *ulam*, which is a type of traditional salad that is normally consumed in raw form by the Asian populations (Southeast Asia, Japan, Korea, and India) with rice or fermented sauces. 7, 8 This traditional dish was not only found to have contained a high level of antioxidants, the polyphenol in the *ulam* was also reported in a review as attenuating the oxidative stress that consequently prevents cognitive decline.^8^ Although the neuropsychological batteries (ie, Mini‐mental State Examination, Digit Span, General Health Questionnaire) had been used for measuring the respective benefits of *Polygonum minus* supplements^9^ as well as the *Centella asiatica* 10 and *Oenanthe javanica* 11 for improving the attention and memory among middle‐aged women and the cognitive impairment associated with the neurodegenerative disorders such as Alzheimer's disease and dementia; however, brain activation using fMRI is yet to be investigated.^12^ As such, the fMRI technique is therefore recommended to assess the working memory performance and cognitive flexibility based on brain activation that is located at the dorsolateral prefrontal cortex of the subjects.

Functional MRI had been used in several studies for determining the relationship between dietary nutrients or vegetables consumption and brain activation^6^, ^13^ such as the association of vegetable consumption and the brain activation that was located at the left superior frontal gyrus of 23 Korean young adults^13^ and the significant relationship between the consumption of vitamin B6 and brain activation of 15 community dwelling adults,^6^ there is still little research known on the benefits of *ulam* consumption among the population with a high risk of cognitive decline. For this reason, this study aimed to examine the association between *ulam* consumption and the working memory and cognitive flexibility of the aging adults from low‐income households through the use of fMRI.

## Materials and Methods

### *Study Design and Sampling*

A cross‐sectional study was conducted on 32 subjects (aged 45–75 years old) that were recruited from the low‐income residential areas of Klang Valley, Malaysia, and were screened earlier for eligibility by basing on the inclusion and exclusion criteria. In this study, the inclusion criteria consisted of adults between the age of 45–75 who are able to speak Malay or English languages, while the exclusion criteria were a history of mental health illness (ie, Alzheimer's disease, schizophrenia, history of stroke), physical disabilities, chronic kidney disease, undergoing dialysis, alcohol and drug users, being claustrophobic, and having internal metallic or electronic implants. The basic health profiles and medical history were obtained by referring to a personal medical book and self‐reported by the subjects. The sample size was calculated using the formula^14^:

$$\begin{array}{l} n={\left[\frac{\left(\mathrm{z\alpha}+\mathrm{z\beta}\right)}{C}\right]}^{2}+3\\ ={\left[\frac{\left(1.96+0.842\right)}{0.61}\right]}^{2}+3\\ =24 \end{array}$$

in which *Zα* = confidence interval 95% = 1.96; *Zβ* = 80% power = 0.842; C = 0.5*ln[(1 + r)/(1‐r)] = 0.61; *r* = correlation coefficient^13^ = 0.55, and additional dropout 20%; thus, the total sample size was 32 subjects. The fMRI scan had been carried out at the selected hospital by a trained fMRI radiographer with ethical approval (NN‐2018‐097) granted from the Institutional Research and Ethics Committee.

### *Procedures*

This study was conducted according to the guidelines as stated in the Declaration of Helsinki 1964. A detailed information sheet that consisted of the purpose of the study, procedures, instructions on completing a required task, and risks was then distributed and explained to the subjects before the attainment of an informed consent. Apart from using the surveys to gauge the sociodemographic background, the trained fieldworkers had also obtained the dietary intake information of the subjects such as the type, amount, cooking method, and the frequency of the common food consumption in the past 7 days through the use of a validated Dietary History Questionnaire (DHQ).^15^ The identification on the types, frequency, and quantity of *ulam* consumption by the subjects was validated using the vegetables and fruits Food Frequency Questionnaire (FFQ) of 58 vegetables, 24 *ulam*, and 26 fruits^16^ were then used as a checklist to complete the DHQ data, where the dietary intake would be analyzed with the Nutritionist ProTM (Axxya Systems, Stafford, TX) and compared against those of the Recommended Nutrient Intake 2017 (RNI) for Malaysians.^17^

This study utilized three validated neuropsychological batteries (ie, Mini‐mental State of Examination,^18^ Digit Span,^19^ and Rey Auditory Verbal Learning Test^20^) in assessing the global cognitive function, working and episodic memory of the subjects, and the scaled Digit Span score was calculated based on the age‐specific tables of the manual in its analysis purpose.^19^

### *fMRI Protocol*

#### *N‐back Task*

**Figure 1:**
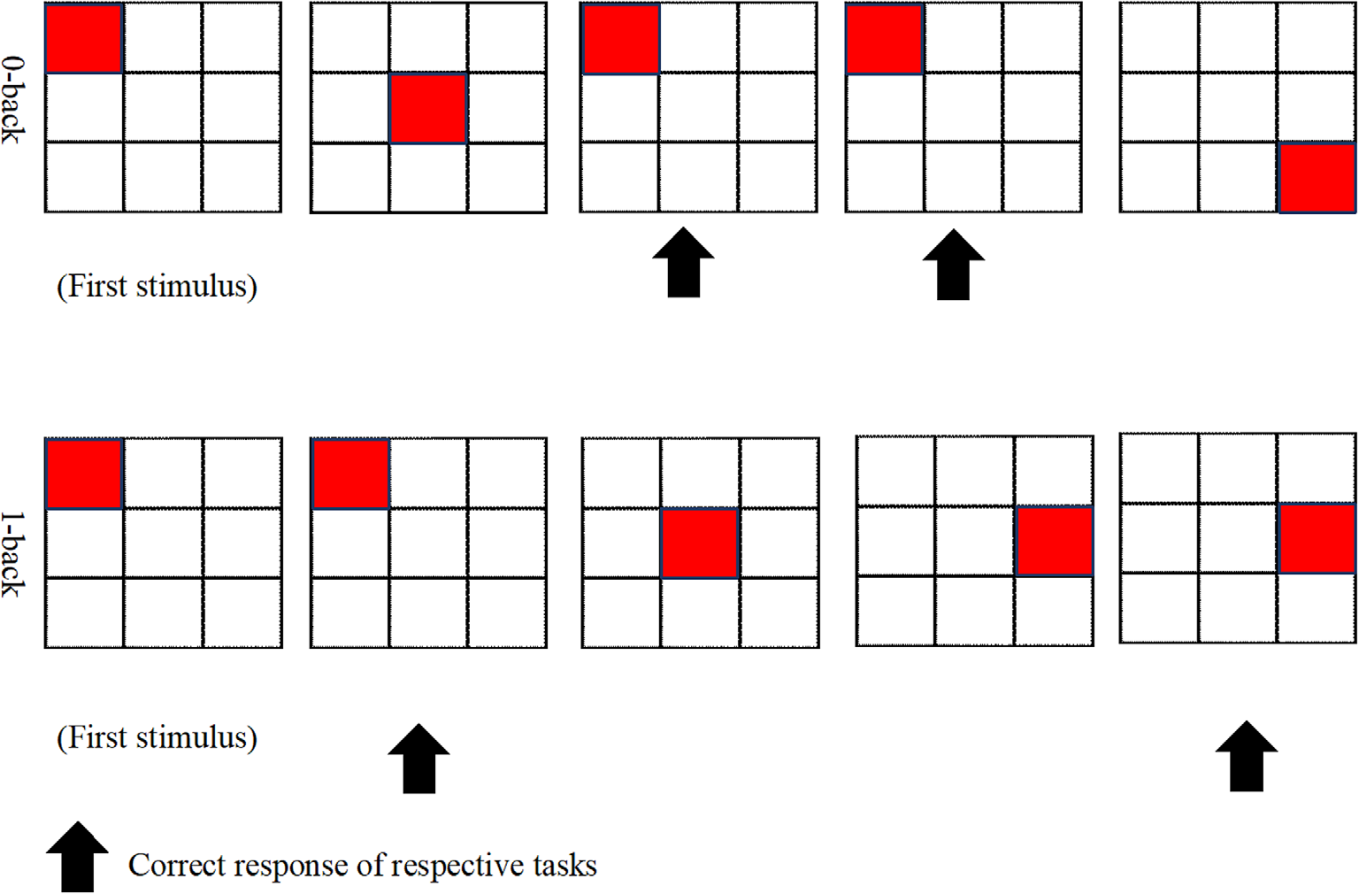
0‐back and 1‐back paradigm.

The two conditions of the N‐back task that were used in this study consisted of the 0‐back and 1‐back that were employed by a previous study,^6^ which had been created and displayed by using the Superlab 5 (Cedrus, San Pedro, CA). There are four blocks for each 0‐back and 1‐back tasks. The duration of each block is 30 seconds; there were 30 seconds rest between blocks and the total duration to complete the task was 510 seconds. Prior to conducting the fMRI scan, a trained fieldworker would demonstrate the completion of the N‐back task by first showing the diagram of the four corresponding 0‐back and 1‐back blocks of each condition (Fig. 1) to the subjects, where they are required to respond to a presented stimulus and to identify if the location of the target had been the same as the initial block (predefined stimulus) under the 0‐back condition and if the target location displayed had been the same as the one preceding it under the 1‐back condition. A trained fMRI radiographer then instigated a 3‐minute anatomical brain scan before proceeding with the 8.5‐minute duration of the N‐back tasks.

#### *Stroop Color Word Test (SCWT)*

**Figure 2:**
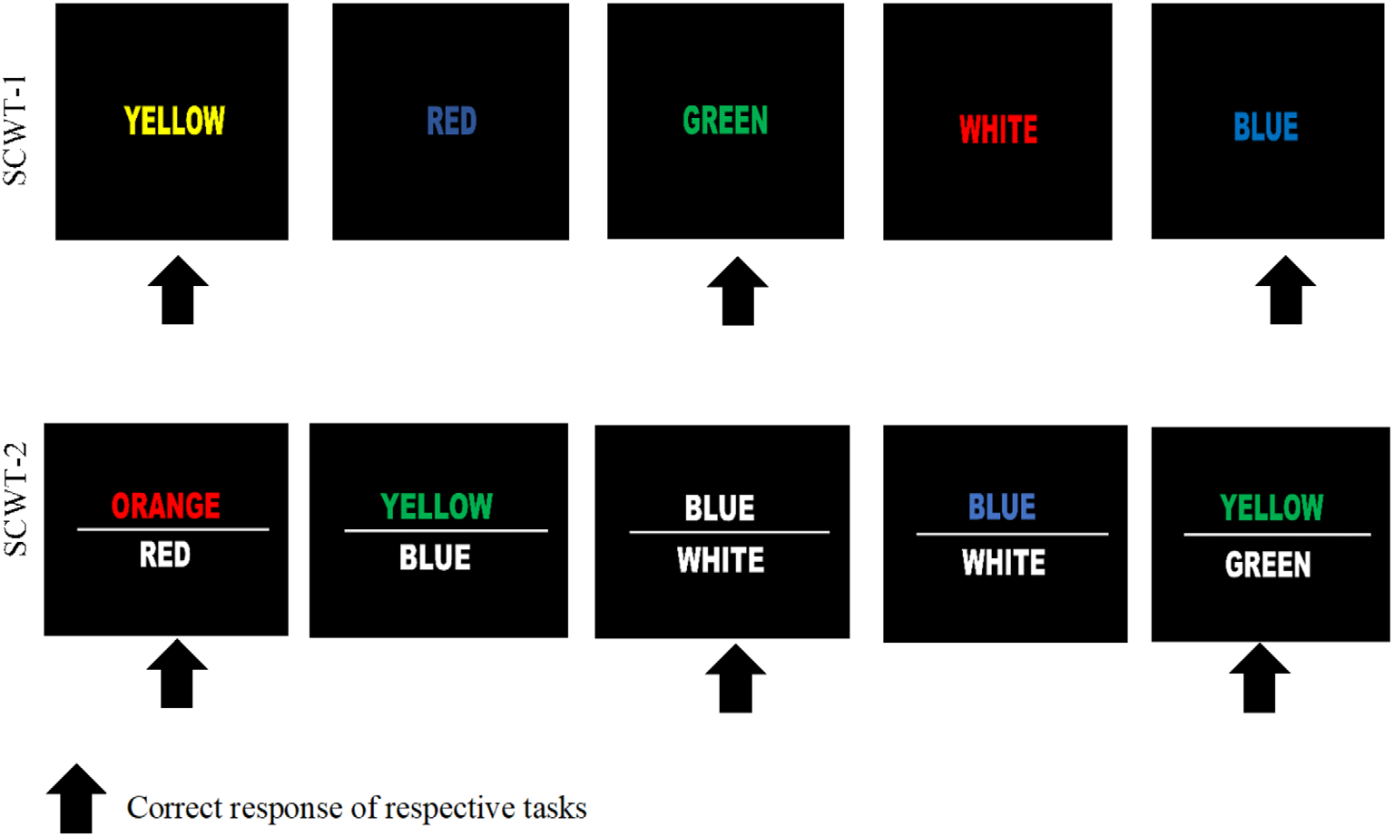
SCWT paradigm.

This task consisted of two paradigms with a respective four task blocks of 15 trials as suggested by a previous study.^21^ There are four blocks for each SCWT‐1 and SCWT‐2 tasks. The duration of each block is 30 seconds; there is 30 seconds rest between blocks and the total duration to complete the task is 510 seconds. In the first paradigm (SCWT‐1), the subjects were initially presented with a series of colored words (white, blue, yellow, green, red), which may be matched accordingly to the word (eg, the word "yellow" is displayed in yellow ink) or had appeared as a different color (eg, the word "red" is displayed in blue ink) and had to indicate the color in which the word is written, hence disregarding the matching of the word itself. In the second paradigm (SCWT‐2), a white colored word would be displayed under a colored word that is printed in different colors, where the meaning of the lower word would sometimes be congruent with the color that was indicated by the upper word (eg, the lower word is written as red, while the upper word "orange" is displayed in red ink) or is different from those of the upper word (eg, the lower word is written as blue, while the upper word "yellow" is displayed in green ink) (Fig. 2) and the subjects were required to indicate if the color of the upper word corresponded with the meaning of the lower word. This task was created and displayed by using Superlab 5 (Cedrus).

### *fMRI Data Acquisition*

Single‐shot spin‐echo echoplanar imaging (EPI) was acquired with the fMRI data and the fMRI images were performed on a 3.0T magnetic resonance (MR) scanner (Magnetom, Trio, Siemens, Erlangen, Germany) with each of the subjects being subjected to high‐resolution T_1_‐weighted anatomical images (repetition time [TR] = 1900 msec, echo time [TE] = 2.35 msec, voxel dimensions 1.0 × 1.0 × 1.0 mm, 250 × 250 voxels, 176 slices, slice thickness = 1 mm), while those of the N‐back task and SCWT were conducted via the T_2_*‐weighted imaging data (TR = 3000 msec, TE = 30 msec, 3 mm isotropic voxels, flip angle = 90°, 27 slices, slice thickness = 4 mm).

### *Behavioral Performance Data*

The percentage of accuracy and the mean response time (RT) on the N‐back and SCWT task of each subjects were then recorded in the calculation of the average data. Correct response (CR) is the percentage of correct responses from total response performed by each subject. A new index $\frac{\text{Response time}}{\text{Correct response}\;}$ was used to analyze the behavioral performance of data.

### *Preprocessing and Functional Imaging Data Analysis*

A quality control test for the MRI machine was done routinely by the professional engineer to make sure that the quality of the functional images was good. The preprocessing and data analysis stage utilized the Statistical Parametric Mapping software implemented in MatLab 9.4.0 R2018a (MathWorks, Natick, MA). The first ten volumes were discarded because of equilibration effects. Functional images were first slice time‐corrected and subjected to a realignment of motion correction according to the mean image of the series. These functional images would then be coregistered to the subject's mean T_1_‐weighted image and estimated against a standardized Montreal Neurological Institute (MNI) stereotaxic space, where the spatial normalization procedure would involve a six‐parameter affine transformation with a spatial transformation matrix. After undergoing the normalization process, all of the functional volumes were then subjected to spatial smoothing with a 6‐mm full‐width half‐maximum of isotropic Gaussian kernel as a way of increasing the signal‐to‐noise level through the removal of the high‐frequency information and the reduction of its intersubject variability.

### *Region of Interest (ROI) Analysis*

The DLPFC that was selected had been defined by the WFU PickAtlas with Brodmann's areas 9 and 46, since prior studies had identified this ROI as being responsible for generating the working memory and cognitive flexibility of the human brain.^6^, ^22^, ^23^ After the individual subject analysis had been performed and corrected significantly (*P* < 0.05, familywise error [FWE]‐corrected), the primary outcome of the ROI brain activation would then be represented by the respective subject's percent of signal change.

### *Statistical Analysis*

All of the statistical analyses were performed using the Statistical Package for the Social Sciences (SPSS, Chicago, IL) v. 23.0 software with a significance level of *P* < 0.05. Apart from utilizing Shapiro–Wilk for testing the normality of the data (*P* > 0.05), the demographic data of all the respondents were also presented in the form of percentages or mean and standard deviation. Pearson correlation was used to analyze the relationship between behavioral performance index and brain activation. While the association between *ulam* consumption and working memory and cognitive flexibility (ie, neuropsychological test scores and DLPFC activation) had been established from the use of a simple linear regression, the adjusted age, gender, and years of formal education were then conducted via the multiple linear regression. β represents the correlation value of the analysis. To control for the inflated FWE rates that results from performing multiple tests on the same data, the significance of these multiple linear regression at a Bonferroni‐adjusted alpha level was performed. To make adjustment, the family‐wise alpha level (0.05) was divided by the total numbers of dependent variables using the formula:

$$P_{\text{corrected}}=\frac{0.05}{12}=0.0042$$

The difference in the neuropsychological test scores and DLPFC activation among the four percentiles of *ulam* consumption as controlled by the age, gender, and years of formal education would be further determined by a univariate analysis of covariance (ANCOVA). The magnitude of *ulam* was taken daily in order to have better working memory and cognitive flexibility that could be obtained from ANCOVA analysis.

## Results

As shown in Table 1, the average of *ulam* consumption was found to be 31.7g/day (~0.5 servings/day), while the average of reaction accuracy and reaction time was 58.7% (N‐back), 70.6% (SCWT), and 2295 (N‐back) and 2495 (SCWT) msec.

**Table 1:** Descriptive Analysis (Expressed in Mean and Standard Deviation)

Parameters	Total subjects (*n =* 32)	Normal range^a,b,c^
Age (years)^a^	57.4 ± 6.7	68
Gender (men, %)^a^	34.4	48
Education (years)^a^	11.2 ± 2.2	8
Household income (USD/month)^a^	800.5 ± 642.1	336
Total *ulam* intake (g/day)^b^	31.7 ± 9.4	40
Total vegetables (non‐*ulam*) intake (g/day)^b^	168.1 ± 61.7	133
Total fruits intake (g/day)^b^	135.9 ± 58.9	179
Dietary nutrients c		
Energy (kcal/day)	1847 ± 315	1550–1920
Protein (g/day)	64.4 ± 11.5	50–61
Carbohydrate (%/day)	56.4 ± 0.9	50–60
Fat (%/day)	29.7 ± 0.7	25–35
Vitamin A (μg/day)	701.3 ± 409.1	600
Vitamin C (mg/day)	87.2 ± 22.7	70
Thiamine (mg/day)	1.2 ± 0.2	1.1–1.2
Riboflavin (mg/day)	1.2 ± 0.3	1.1–1.3
Niacin (mg/day)	14.8 ± 2.7	14–16
Sodium (mg/day)	2073.5 ± 434.8	1500
Potassium (mg/day)	2463.3 ± 507.5	4700
Calcium (mg/day)	459.2 ± 188.7	1000–1200
Iron (mg/day)	19.3 ± 5.4	11–14
Phosphorus (mg/day)	832.3 ± 108.5	700
Neuropsychological batteries		
MMSE	28.7 ± 2.3	23.8
Digit span (scaled score)	10.1 ± 1.7	7.7
RAVLT immediate recall	36.6 ± 9.2	37.8
RAVLT delayed recall	8.7 ± 2.6	7.7
fMRI behavioral performance		
Reaction accuracy (N‐back) (%)	58.7 ± 14.9	N/A
Reaction time (N‐back) (msec)	2295 ± 429	N/A
Reaction accuracy (SCWT) (%)	70.6 ± 8.4	N/A
Reaction time (SCWT) (msec)	2492 ± 331	N/A
fMRI brain activation		
0‐back total percent signal change (%) Left DLPFC	0.51 ± 0.23	N/A
0‐back total percent signal change (%) Right DLPFC	0.68 ± 0.19	N/A
1‐back total percent signal change (%) Left DLPFC	0.49 ± 0.22	N/A
1‐back total percent signal change (%) Right DLPFC	0.69 ± 0.20	N/A
SCWT‐1 total percent signal change (%) Left DLPFC	0.78 ± 0.40	N/A
SCWT‐1 total percent signal change (%) Right DLPFC	0.90 ± 0.43	N/A
SCWT‐2 total percent signal change (%) Left DLPFC	0.91 ± 0.44	N/A
SCWT‐2 total percent signal change (%) Right DLPFC	1.05 ± 0.46	N/A

a Nationwide aging population research in Malaysia.^38^

b Vegetables and fruits intake among population in Malaysia.^39^

c Recommended Nutrient Intake for Malaysian population.^17^

DLPFC, dorsolateral prefrontal cortex; RAVLT, Rey Auditory Verbal Learning Test; fMRI, functional magnetic resonance imaging.

Table 2 shows the correlation between the behavioral performance index and brain activation. Significant negative correlations were observed between the behavioral performance index and right DLPFC activation and on 0‐back (*r* = –0.456, *P* = 0.009), 1‐back (*r* = –0.484, *P* = 0.005), and left DLPFC activation while subjects performed SCWT‐1 (*r* = –0.353, *P* < 0.05) and SCWT‐2 (*r* = –0.356, *P* < 0.05).

**Table 2:** Correlation Between Behavioral Performance Index and DLPFC Activation

Brain activation	Behavioral performance index (RT/CR)	
	Correlation (*r*)	*P‐*value
0‐back total percent signal change (%) Left DLPFC	–0.139	0.448
0‐back total percent signal change (%) Right DLPFC	–0.456*	0.009
1‐back total percent signal change (%) Left DLPFC	–0.141	0.441
1‐back total percent signal change (%) Right DLPFC	–0.484*	0.005
SCWT‐1 total percent signal change (%) Left DLPFC	–0.353*	0.047
SCWT‐1 total percent signal change (%) Right DLPFC	–0.129	0.482
SCWT‐2 total percent signal change (%) Left DLPFC	–0.356*	0.046
SCWT‐2 total percent signal change (%) Right DLPFC	–0.062	0.735

Significant at *P <* 0.05.

CR: correct response; DLPFC, dorsolateral prefrontal cortex; RT: response time; SCWT, Stroop Color Word Test.

**Figure 3:**
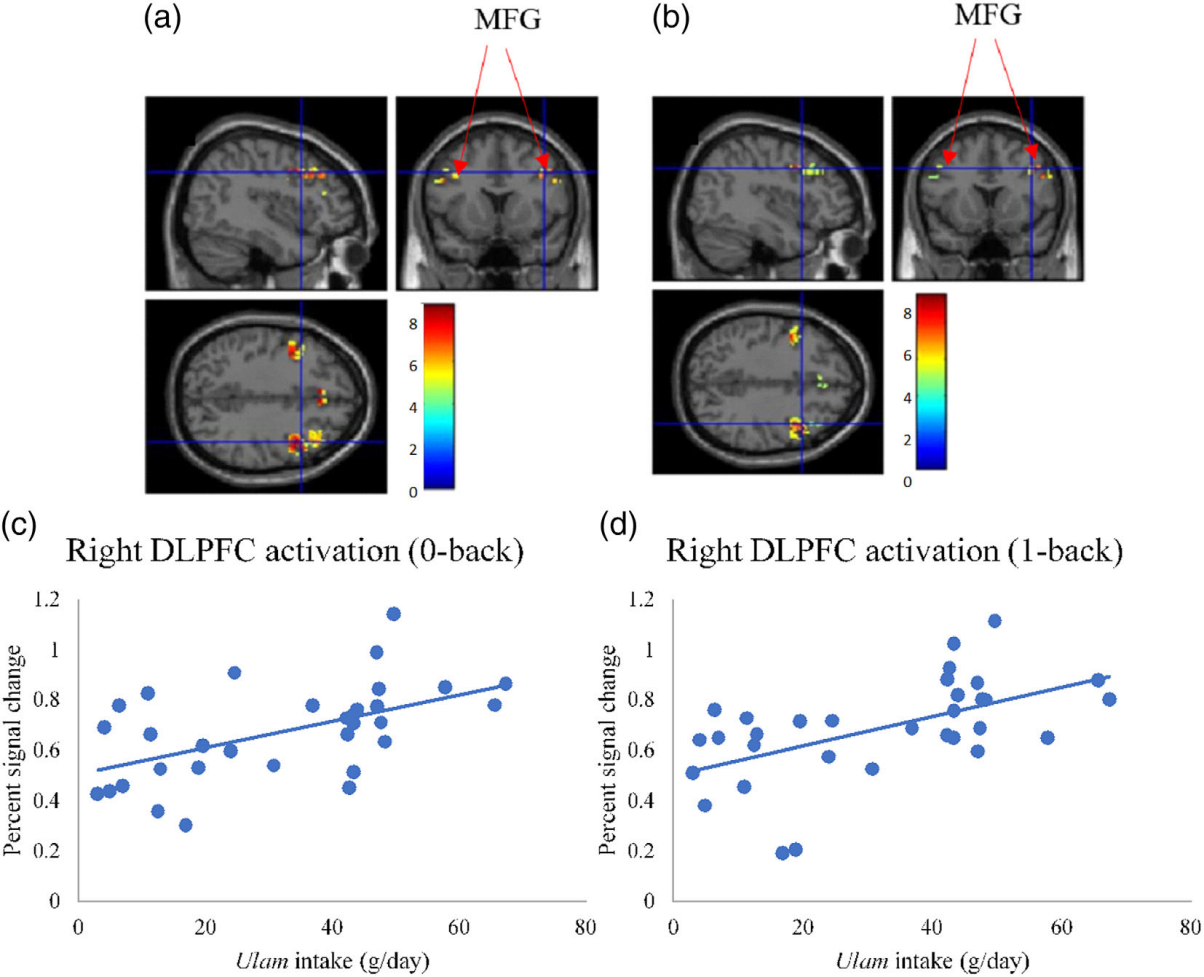
Association between *ulam* intake and brain activation. **(a)** Activated brain region in DLPFC when performing the 0‐back task (*P* < 0.05, FWE‐corrected). **(b)** Activated brain region in DLPFC when performing the 1‐back task (*P* < 0.05, FWE‐corrected). **(c,d)** Scatterplots of positive association between *ulam* intake and DLPFC activation. FWE: familywise error; DLPFC, dorsolateral prefrontal cortex.

**Figure 4:**
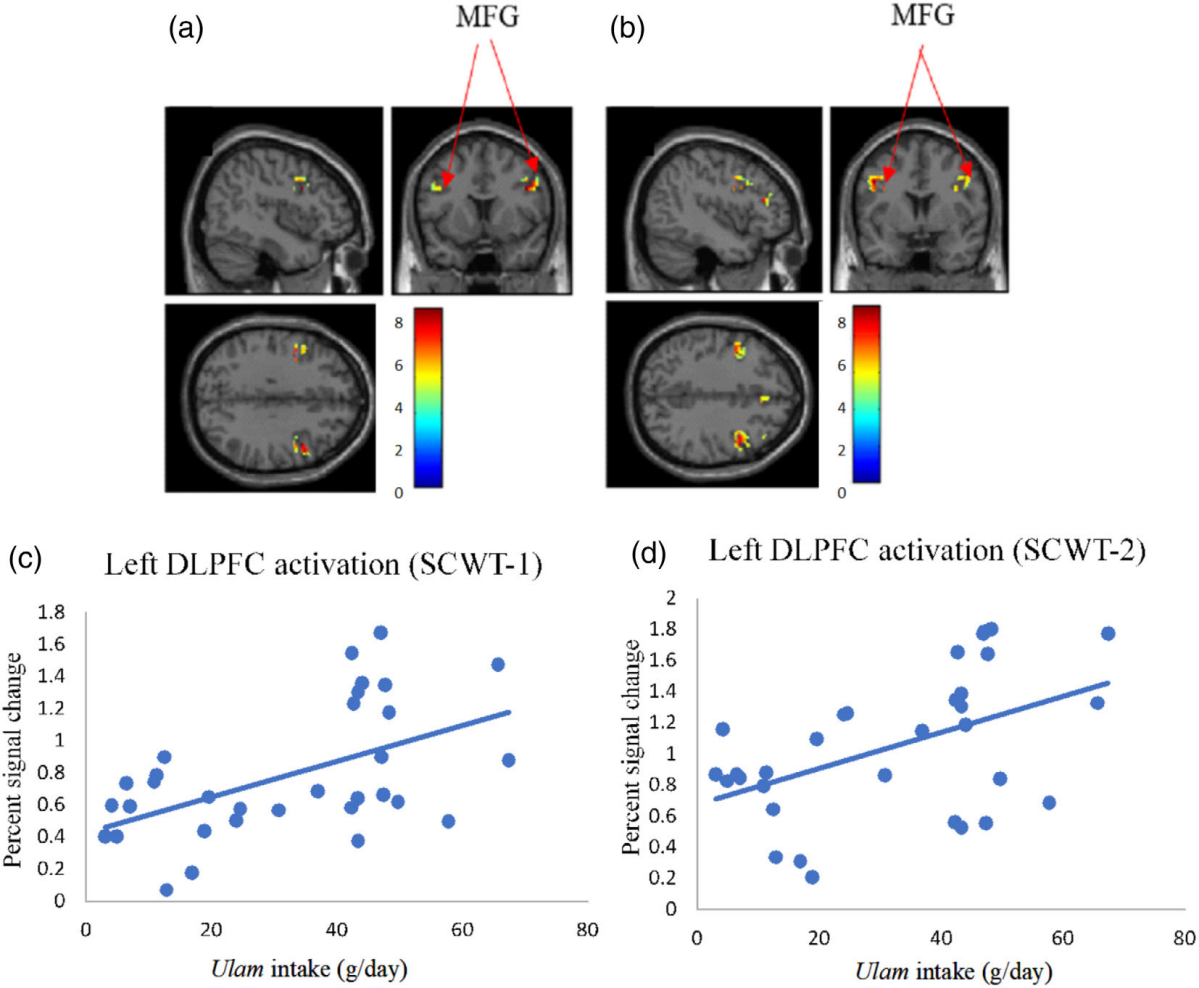
Association between *ulam* intake and brain activation. **(a)** Activated brain region in DLPFC when performing SCWT‐1 (*P* < 0.05, FWE‐corrected). **(b)** Activated brain region in DLPFC when performing SCWT‐2 (*P* < 0.05, FWE‐corrected). **(c,d)** Scatterplots of positive association between *ulam* intake and DLPFC activation. FWE: familywise error; DLPFC, dorsolateral prefrontal cortex; MFG, middle frontal gyrus.

Apart from demonstrating a correlation between *ulam* consumption and the Digit Span (R^2^ = 0.45, β = 0.672, *P* < 0.001), the univariate analysis also showed that *ulam* consumption was correlated with the right DLPFC activation (0‐back) (R^2^ = 0.28, β = 0.533, *P* = 0.002), right DLPFC activation (1‐back) (R^2^ = 0.31, β = 0.556, *P* = 0.001), left DLPFC activation (SCWT‐1) (R^2^ = 0.27, β = 0.523, *P* = 0.002) and the left of DLPFC activation (SCWT‐2) (R^2^ = 0.24, β = 0.490, *P* = 0.004) (Table 3). The use of multiple linear regression had also shown the relationship of *ulam* consumption as being specifically significant with Digit Span (R^2^ = 0.51, β = 0.702, *P* < 0.001), right DLPFC activation (1‐back) (R^2^ = 0.34, β = 0.591, *P* = 0.001), left DLPFC activation (SCWT‐1) (R^2^ = 0.33, β = 0.553, *P* = 0.002), and left DLPFC activation (SCWT‐2) (R^2^ = 0.34, β = 0.497, *P* = 0.004) after the age, gender, and years of formal education adjustments (Table 3). The relationship between *ulam* consumption and DLPFC activation while subjects performed N‐back and SCWT is shown in Figs. 3 and 4.

**Table 3:** Association Between *Ulam* Consumption With Cognitive Function and Brain Activation

Variables	*Ulam* consumption					
	Simple linear regression			Multiple linear regression		
Neuropsychological batteries and brain activation	R^2^	Adjusted odd ratio (95% CI)	*P‐*value	R^2^	Adjusted odd ratio (95% CI)	*P‐*value
MMSE	0.04	0.207 (–0.019–0.068)	0.257	0.10	0.233 (–0.018–0.073)	0.225
Digit span	0.45	0.672 (0.035–0.083)*	<0.001	0.51	0.702 (0.038–0.099)*	<0.001
RAVLT (total immediate recall)	0.20	0.446 (0.057–0.395)	0.010	0.32	0.439 (0.054–0.391)	0.012
RAVLT (delayed recall)	0.15	0.382 (0.004–0.099)	0.034	0.37	0.422 (0.013–0.101)	0.013
0‐back right DLPFC activation	0.28	0.533 (0.002–0.008)*	0.002	0.30	0.511 (0.002–0.008)	0.005
0‐back left DLPFC activation	0.07	0.266 (–0.001–0.008)	0.142	0.10	0.233 (–0.002–0.008)	0.226
1‐back right DLPFC activation	0.31	0.556 (0.003–0.009)*	0.001	0.34	0.591 (0.003–0.010)*	0.001
1‐back left DLPFC activation	0.08	0.280 (–0.001–0.007)	0.120	0.09	0.284 (–0.001–0.008)	0.144
SCWT‐1 right DLPFC activation	0.11	0.333 (0.000–0.015)	0.062	0.16	0.348 (–0.001–0.016)	0.066
SCWT‐1 left DLPFC activation	0.27	0.523 (0.004–0.018)*	0.002	0.33	0.553 (0.005–0.019)*	0.002
SCWT‐2 right DLPFC activation	0.08	0.287 (–0.002–0.015)	0.112	0.19	0.317 (–0.001–0.016)	0.085
SCWT‐2 left DLPFC activation	0.24	0.490 (0.004–0.019)*	0.005	0.34	0.497 (0.004–0.020)*	0.004

* Significant at *P <* 0.0042.

MLR model was adjusted by age, gender and years of formal education.

CI, confidence interval; DLPFC, dorsolateral prefrontal cortex; fMRI, functional magnetic resonance imaging; RAVLT, Rey Auditory Verbal Learning Test; SCWT, Stroop Color Word Test.

A further analysis from the univariate ANCOVA is shown in Table 4 with the age, gender, and years of formal education adjustments, which also discovered the *ulam* consumption at the 100^th^ percentile as having a significantly higher Digit Span score (*P* < 0.001, f = 1.06) and Rey auditory verbal learning test (RAVLT) (total immediate recall) (*P* < 0.05, f = 0.67) than those of the 25^th^ percentile's, while the *ulam* consumption at the 100^th^ and 75^th^ percentile demonstrated a significantly higher right DLPFC activation for the 0‐back condition (*P* < 0.05, f = 0.61), 1‐back condition (*P* < 0.01, f = 0.85), SCWT‐1 condition (*P* < 0.05, f = 0.90), and the SCWT‐2 condition (*P* < 0.05, f = 0.65) as compared with the 25^th^ and 50^th^ percentiles.

**Table 4:** Univariate Analysis of Covariance (ANCOVA) Between *Ulam* Consumption and Cognitive Parameters

Variables	*Ulam* consumption				*P‐*value	Effect size Cohen's f
Cognitive parameters	25^th^ percentile (<13g) (*n =* 8)	50^th^ percentile (13‐37g) (*n =* 8)	75^th^ percentile (38‐46g) (*n =* 8)	100^th^ percentile (≥47g) (*n =* 8)		
MMSE	27.25 ± 3.33	29.75 ± 0.46	29.88 ± 0.35	28.13 ± 2.42	0.332	0.70
Digit span	8.88 ± 0.64^a^	8.87 ± 0.99^b^	10.63 ± 1.85	11.63 ± 1.19^ab^	<0.001	1.06
RAVLT (total immediate recall)	27.38 ± 6.28^abc^	40.50 ± 8.60^a^	41.75 ± 7.21^b^	39.13 ± 10.00^c^	0.024	0.67
RAVLT (delayed recall)	6.88 ± 1.73	8.88 ± 1.81	10.29 ± 3.04	9.00 ± 2.98	0.127	0.52
0‐back right DLPFC activation	0.58 ± 0.18^a^	0.60 ± 0.18^b^	0.69 ± 0.16	0.82 ± 0.15^ab^	0.048	0.61
0‐back left DLPFC activation	0.52 ± 0.25	0.39 ± 0.19	0.45 ± 0.20	0.67 ± 0.23	0.070	0.56
1‐back right DLPFC activation	0.59 ± 0.13^ac^	0.24 ± 0.22^bd^	0.82 ± 0.13^ab^	0.79 ± 0.16^cd^	0.003	0.85
1‐back left DLPFC activation	0.48 ± 0.15	0.40 ± 0.27	0.49 ± 0.14	0.59 ± 0.29	0.446	0.33
SCWT‐1 right DLPFC activation	0.84 ± 0.21	0.85 ± 0.50	0.93 ± 0.43	1.19 ± 0.39	0.131	0.50
SCWT‐1 left DLPFC activation	0.65 ± 0.18^a^	0.46 ± 0.22^bc^	1.09 ± 0.49^ab^	0.95 ± 0.36^c^	0.002	0.90
SCWT‐2 right DLPFC activation	0.83 ± 0.46	0.72 ± 0.49	1.02 ± 0.38	1.06 ± 0.44	0.229	0.43
SCWT‐2 left DLPFC activation	0.86 ± 0.14^b^	0.81 ± 0.45^ac^	1.21 ± 0.45^a^	1.30 ± 0.53^bc^	0.030	0.65

^a, b, c, d^Significant at *P <* 0.05 using least significance difference (LSD) post‐hoc test.

The model was adjusted by age, gender, and years of formal education.

CI, confidence interval; DLPFC, dorsolateral prefrontal cortex; fMRI, functional magnetic resonance imaging; RAVLT, Rey Auditory Verbal Learning Test; SCWT, Stroop Color Word Test.

Meanwhile, the activated brain region in DLPFC (Brodmann's areas 9 and 46) when performing the N‐back task (*P* < 0.05, FWE‐corrected) as depicted in Table 5 and Figs. 3 and 4. The total voxels activated in DLPFC for N‐back and SCWT was 1081 and 773 voxels, respectively, with the highest activation observed in the middle frontal gyrus for all of the N‐back and SCWT conditions (*P* < 0.05, FWE‐corrected) and the other activated regions were observed in the superior frontal gyrus, inferior frontal gyrus, and precentral gyrus.

**Table 5:** Activated Brain Regions When Performing SCWT (*P <* 0.05, FWE Corrected)

Anatomical region	L/R	Coordinates x,y,z	Voxels activated	Maximum T value
0‐back				
Middle frontal gyrus	L	–40 2 32	99	8.61
Inferior frontal gyrus	R	44 10 30	324	8.53
Superior frontal gyrus	R	4 30 38	37	8.05
	L	–4 30 38	24	8.02
Middle frontal gyrus	L	–46 12 30	40	7.83
	L	–36 4 32	3	7.05
	R	46 30 24	17	6.38
	R	34 42 30	5	5.54
	L	–40 36 20	7	5.51
	R	44 38 24	4	5.22
Precentral gyrus	R	38 4 32	6	5.16
Middle frontal gyrus	L	–34 32 34	2	4.97
Inferior frontal gyrus	R	54 26 14	2	4.93
1 back				
Middle frontal gyrus	L	–40 2 32	130	9.50
	R	48 20 28	258	9.07
	R	46 30 22	28	6.65
	R	38 4 32	6	6.54
	L	–36 4 32	3	6.48
	R	46 32 34	30	6.12
Superior frontal gyrus	R	10 26 36	20	6.07
	L	–4 30 36	14	6.06
Middle frontal gyrus	L	–42 38 24	3	5.29
	L	–52 22 28	11	5.22
	L	–44 16 28	4	5.03
	L	–32 42 30	1	4.81
	R	34 42 30	1	4.79
	R	32 36 30	1	4.73
Superior frontal gyrus	R	4 34 34	1	4.64
SCWT‐1				
Middle frontal gyrus	R	44 10 32	148	9.73
	L	–40 2 32	51	9.29
	L	–42 32 18	45	7.82
	L	–36 4 32	3	7.61
	L	–46 10 32	24	6.59
	L	–46 20 26	8	5.98
	R	48 40 22	1	5.88
	R	38 4 32	1	5.07
	R	46 32 24	1	4.89
Superior frontal gyrus	R	6 34 38	4	4.88
SCWT‐2				
Middle frontal gyrus	L	–46 4 36	164	9.61
	R	50 10 36	176	9.12
	L	–42 3 18	68	8.37
	L	–36 4 32	3	8.07
	R	42 34 36	18	7.11
Superior frontal gyrus	R	8 28 36	36	7.10
Middle frontal gyrus	R	38 4 30	6	6.91
	R	44 36 20	11	6.02
	R	48 32 24	2	5.21
	L	–44 20 34	2	5.02
	L	–44 32 34	1	4.90

L: left, R: right.

## Discussion

Our findings demonstrated that high *ulam* consumption was related to greater right DLPFC activation while the subjects were performing the N‐back task. 0‐back and 1‐back tasks were designed to assess the cognitive functions like attention, working memory, and short and long‐term memory in middle‐age and older age. The N‐back task has face validity as a working memory task since it seems to require maintaining continuous updating and processing of information.^24^ These findings are also been supported by a previous study in which a significant improvement was reported in the executive functioning and working memory of 20 healthy Malaysian participants after 3 weeks of being supplemented with the *Superulam* capsules (a combination of *ulam* or traditional Asian vegetables extracts).^25^ Another systematic review on the common *ulam, Centella asiatica*, was also reported in Thailand, where the herb was not only discovered for improving working memory and alertness, but also in relieving anger as well.^10^

The high level of antioxidants and polyphenols in *ulam* 26 was also found to be a major contributor to the cognitive pathway, where the latter is known for attenuating apoptosis, removing reactive oxygen species, preventing oxidative stress that causes cognitive decline,^8^ as well as initiating the activation of sirtuins in the regulation of the human body cells that alleviates the occurrence of neurodegenerative illnesses.^27^ Also, the flavonoid from the plants would scavenge for free radicals and decrease the neuron apoptosis that impedes neuroinflammation.^28^ The association of the *ulam* metabolites with the improvement of working memory, however, is yet to be elucidated by future research.

This study had indicated that a high *ulam* consumption resulted in the better cognitive processing strategies, adaptation and flexibility,^29^ an activity that is closely related to DLPFC involvement.^30^ For this reason, although it had seemed that the flavonoids from the *ulam* had entered a specific brain area (ie, DLPFC) and led to the improved cognitive flexibility, further randomized control trials and pharmacological studies would still be required to validate this presumption. The effect size of the ANCOVA analysis from this study was also considerably large,^31^ where the subjects with higher percentiles of *ulam* consumption (at least ½ servings per day) had shown substantially higher Digit Span and RAVLT test scores and greater DLPFC activation than those with lower percentiles of *ulam* consumption.

Another main finding of this study was the highest DLPFC activation that was found in the middle frontal gyrus during the fMRI scan as well as the other activated regions that had appeared in the precentral, superior frontal, and inferior frontal gyrus. While the attention, working memory, and executive functioning are controlled by the prefrontal cortex that is located at the frontal part of the brain,^32^ the working memory, on the other hand, was reported to have been supported by the left superior frontal, right middle frontal gyrus, and the inferior frontal gyrus.^33^ The involvement of the middle frontal gyrus in the working memory tasks such as word‐reading and numerical operations has also been proven from previous studies.^6^, ^33^ Since our findings had shown the significance of right DLPFC activation from performing the N‐back tasks, we can therefore surmise that the subjects with higher *ulam* consumption as demonstrating a greater right‐hemispheric dominance during the visual‐spatial processing stage as compared with lower *ulam* consumption.^34^

As the involvement of the middle frontal gyrus while performing the SCWT had been proven from a previous study,^23^ our findings too had revealed that the subjects with higher *ulam* consumption as demonstrating better left DLPFC activation for both of the SCWT tasks, with the left hemisphere displaying more of the brain activity during the assigned word recognition task. The latest evidence therefore reported the left hemisphere of the human brain as being more affected by the interference than the right hemisphere when naming the color of a word that conflicted with the displayed word (eg, the word "yellow" printed in "orange"). Since the left DLPFC had been selectively activated during the high conflict of the SCWT trials,^35^ this had thus demonstrated a higher left DLPFC activation through the coordinated recruitment of brain systems and, consequently, a higher cognitive skill was observed among the subjects with higher *ulam* consumption.^36^ Although the right DLPFC activation had not been significantly associated with *ulam* consumption, its activation could have been due to the macro‐adjustments of the cognitive control, which minimized the subsequent attentional conflict during the frequent congruent and incongruent SCWT.^23^

The sample size of this study was also calculated and justified sufficiently, where the investigation on the association between *ulam* consumption and brain activation had been successfully conducted through the use of the fMRI; hence, corresponding to several previous studies that were conducted on the nutrients intake and brain activation with a small sample (*n* = 15–30).^6^, ^13^, ^37^ The neuroimaging approach that was complemented with the neuropsychological batteries for the evaluation of cognitive domains such as working memory and cognitive flexibility can thus be regarded as a strength of this investigation, since the variations in brain functions that arose from the initial signs of Alzheimer's disease could be examined by this sort of MRI.

The main limitation of this study was its inability for elucidating the cause–effect relationship between *ulam* consumption and brain activation from a cross‐sectional study design, randomized control trials through the use of fMRI, and the metabolomic approach are therefore recommended to further determine the efficacy of *ulam* for improving brain activation as well as to gauge a better understanding of its neuroprotective properties. In addition, we analyzed the brain activation using ROI analysis (ie, DLPFC); however, whole‐brain analysis is recommended to investigate the *ulam* consumption related to other activated brain regions in future studies.

In conclusion, this study found that high *ulam* consumption was related to a high intensity of brain activation in DLPFC using the fMRI technique. However, there is a need to adopt whole‐brain analysis and randomized controlled trials to investigate the role of *ulam* as a neuroprotective agent affirmatively.
